# Biomimetic Virus-Like Mesoporous Silica Nanoparticles Activate NK Cells Indirectly via Monocyte Crosstalk

**DOI:** 10.1021/acs.nanolett.6c01832

**Published:** 2026-08-27

**Authors:** Minna Sivonen, Silja Saarela, Jiajia Wang, Mira Saari, Emmi Järvelä, Lotta Andersson, Enkhzaya Batnasan, Leena Latonen, Helka Göös, Vesa-Pekka Lehto, Wujun Xu

**Affiliations:** † Department of Technical Physics, University of Eastern Finland, Yliopistonranta 8, 70211 Kuopio, Finland; ‡ Institute of Biomedicine, University of Eastern Finland, Yliopistonranta 8, 70211 Kuopio, Finland; § iCell, Research and Development, 99296 Finnish Red Cross Blood Service, Haartmaninkatu 8, 00290 Helsinki, Finland; ∥ A.I. Virtanen Institute for Molecular Sciences, University of Eastern Finland, Neulaniementie 2, 70210 Kuopio, Finland

**Keywords:** natural killer cells, monocytes, mesoporous silica, virus-like nanoparticles, cancer immunotherapy

## Abstract

Cancer immunotherapies show clinical promise but often rely on T-cell priming and are limited by tumor heterogeneity and the immunosuppressive tumor microenvironment (TME). Innate immune activation offers a complementary approach, with specific aim in natural killer (NK) cell activation for antigen-independent response. Biomimetic nanoparticles combining virus-like morphology with cell membrane (CM) coating offer a strategy to engage this innate immune axis. This study investigates virus-like mesoporous silica nanoparticles (VLPSi) with tunable spikes, surface functionalization, and CM coating as innate immunity modulators. Optimization revealed that longer spikes, amine functionalization, and CM coating synergistically enhance NK cell activation within human PBMCs, as indicated by CD69/CD25 upregulation and IFN-γ secretion. CD14^+^ monocyte depletion attenuated activation, identifying monocyte-dependent crosstalk as a key mechanism. In purified NK cells, engineered CM-coated VLPSi induced early activation and supported feeder-free expansion. These results define topology, surface chemistry, and CM coating as parameters for innate immune modulation.

## Graphical abstract

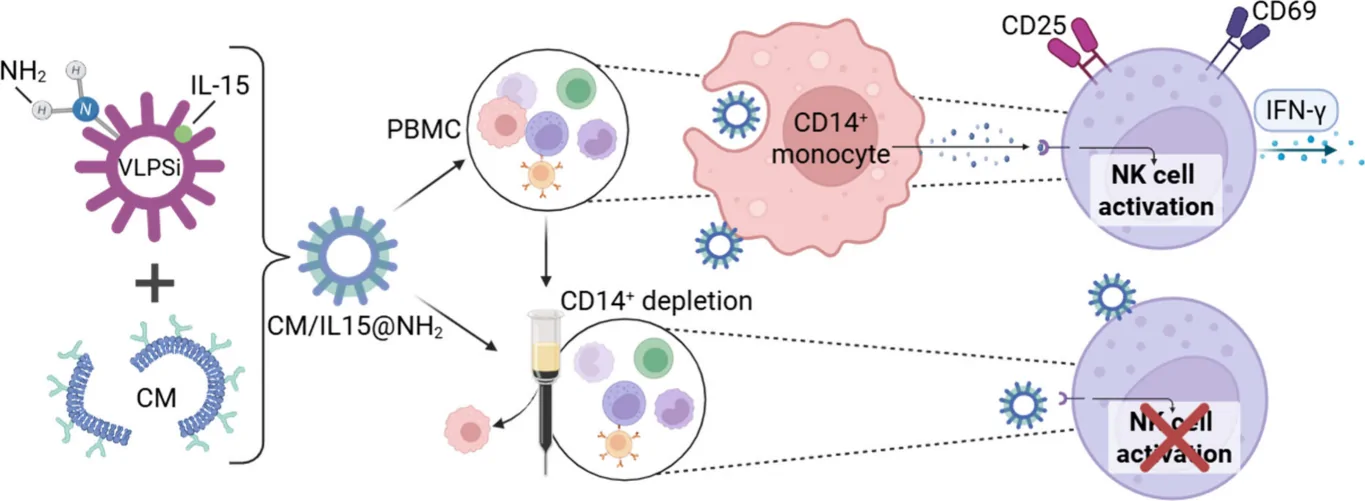

Recent advances in cancer immunotherapy have transformed the therapeutic landscape. Approaches such as immune checkpoint inhibitors,^1^ adoptive cell therapies,^2^ and cancer vaccines^3,4^ harness the patients’ immune system to eliminate malignant cells. However, many of these strategies rely on antigen-specific T-cell priming and cannot overcome tumor heterogeneity and patient variability, resulting in modest response rates and short-term therapeutic benefits.^5^ Therapies that activate the innate immune response, particularly natural killer (NK) cells, do not rely on recognition of a single antigen and therefore hold potential for broader antitumor responses.^6^ NK cells have demonstrated efficacy against hematological tumors, but their effector function and infiltration in solid tumors are often suppressed by the immunosuppressive tumor microenvironment (TME). In addition, interactions with other immune cells have an impact on their activation. For example, macrophages prime NK cells via cytokine signaling (e.g., IL-12 and IL-18) and direct cell–cell contact.^7^ This crosstalk represents an important aspect of innate immune activation and offers mechanisms that could be used in nanomaterial design for cancer immunotherapies.

Nanoparticle (NP) designs that combine activating ligands and cytokine presentation could offer a strategy to mimic NK-cell-activating signals and trigger robust innate immune responses. NPs engineered with biomimetic features, including virus-like surface morphology and biological components, such as cell membrane (CM) coating, offer a versatile platform to integrate synthetic nanomaterials with biologically relevant cues for cancer immunotherapies.^8,9^ Virus-like surface topology can enhance immune activation by mimicking pathogen-associated structural cues, promoting uptake by antigen-presenting cells (APCs), and stimulating innate immune responses.^10^ In parallel, CM coating transfers surface proteins, adhesion molecules, and immunologically relevant ligands from their parent cells onto the NPs.^11,12^ Dependent upon the parent cell, CM coating can be utilized for homotypic targeting, prolonged circulation, and improved biocompatibility.^13^ CMs derived from cancer cells have shown potential to influence NK cell responses through cancer-specific antigen presentation.^8^ Although virus-like nanostructures and CM-coated spherical nanoparticles have independently demonstrated promise for cancer immunotherapy, their combined effects remain largely underexplored. Given that the innate immune response relies on rapid, coordinated communication between myeloid cells and NK cells, cancer immunotherapies capable of engaging this axis could offer significant advantages. This represents an opportunity to design NPs that can more effectively modulate the innate immune response.

In this study, virus-like mesoporous silica nanoparticles (VLPSi) were engineered with rigid tunable spikes and a biomimetic cancer CM coating. Mesoporous silica is widely used for biomedical applications due to its biocompatibility, structural stability, and high drug-loading capacity.^14^ Advances in nanotechnology enable precise control over its particle size, surface morphology, and chemistry.^15,16^ Virus-like surface topology is known to enhance cellular uptake and can stimulate innate immune responses by mimicking pathogen-associated structural cues.^17,18^ Although direct evidence for NK cell activation by virus-like NPs is limited, these cues can engage in innate immune pathways that support NK cell function.^19^ We aimed to investigate how VLPSi morphology, surface functionalization, CM source, and cytokine incorporation influence NK cell activation within human peripheral blood mononuclear cells (PBMCs). These results reveal how NP design features can be utilized to harness innate immune interactions to drive NK cell activation, establishing design principles for innate-immune-engaging NPs for next-generation cancer immunotherapies.

VLPSi with approximately 5 nm (VLPSi-5) and 30 nm (VLPSi-30) virus-like spikes were synthesized using a single-micelle epitaxial growth approach. VLPSi with 30 nm spikes have previously demonstrated higher cellular internalization and immune engagement^19^ and were selected as the primary platform for subsequent studies, while VLPSi-5 were used as a reference control (Figure S3A).

The influence of spike length and CM coating to cellular uptake was assessed using FITC-labeled VLPSi-5 and VLPSi-30. CMs derived from IFN-γ-treated MDA-MB-231 (MDA) cells (γCM) served as a source of tumor-associated antigens and biologically relevant ligands. VLPSi and γCM-coated VLPSi (γCM@VLPSi) were incubated with human breast cancer cells (MDA), monocytes (THP-1), and expanded primary human NK cells (exNK). Flow cytometry results revealed that the uptake profile was cell-type-dependent and enhanced by both γCM coating and longer spikes, with γCM@VLPSi-30 showing the highest uptake (Figure 1A). MDA cells had the highest uptake profile, followed by THP-1, while exNK cells showed minimal uptake, with a modest increase observed for γCM@VLPSi-30. Based on these results, VLPSi-30 were selected for subsequent studies.

**Figure 1:**
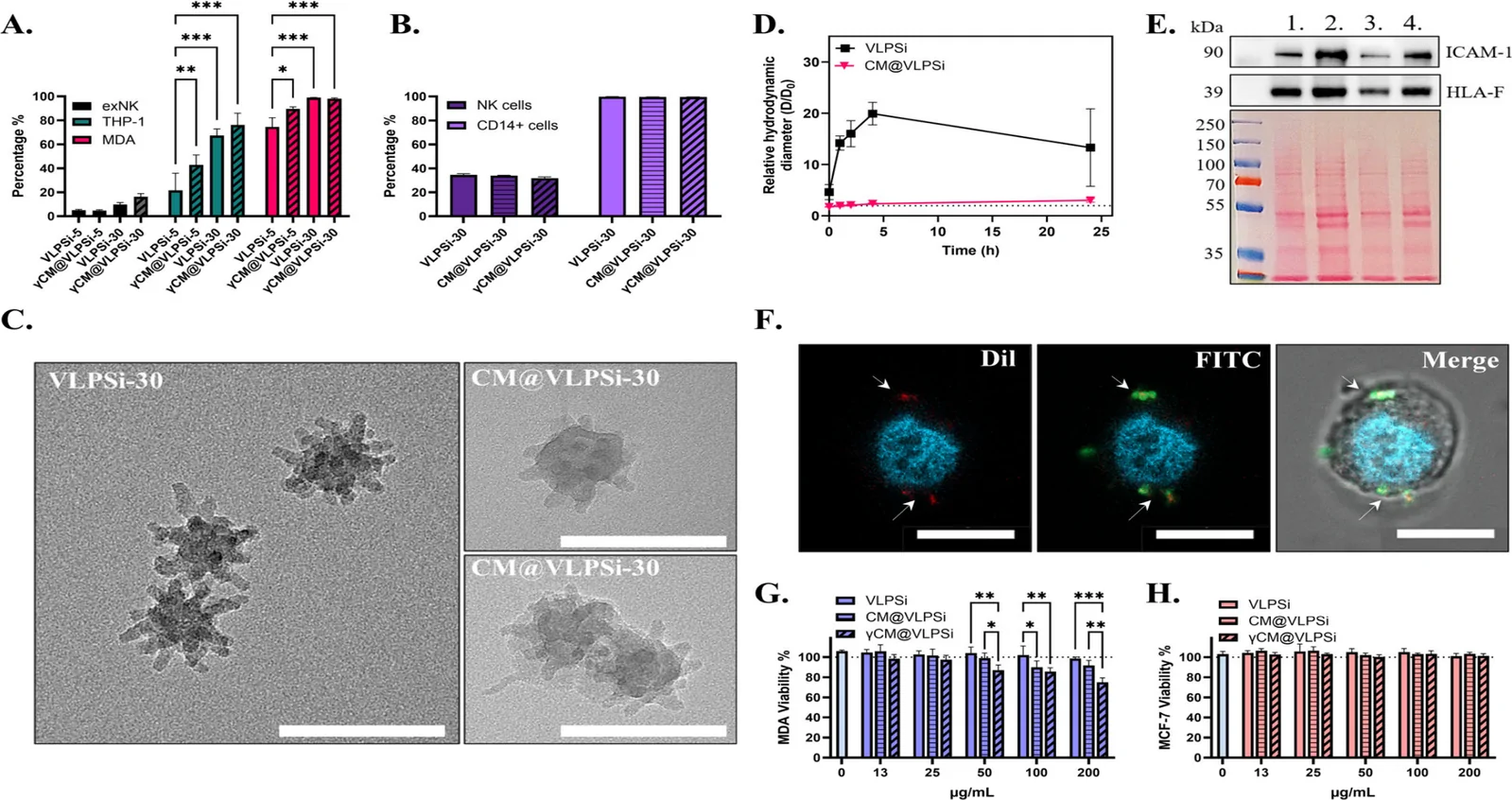
Characterization of virus-like mesoporous silica nanoparticles (VLPSi). (A) Percentage of FITC positive expanded NK cells (exNK), THP-1, and MDA-MB-231 (MDA) cells after 4 h of incubation with FITC-labeled VLPSi carrying 5 nm (VLPSi-5) or 30 nm (VLPSi-30) spikes, with and without IFN-γ-treated MDA cell membrane (γCM) coating (γCM@VLPSi-5 and γCM@VLPSi-30). (B) Percentage of FITC positive naïve NK cells and CD14^+^ cells within PBMC after 4 h of incubation with FITC-labeled VLPSi-30 with and without CM coating. CMs were derived from MDA (CM; striped) and IFN-γ-stimulated MDA (γCM; diagonal pattern). (C) TEM images from VLPSi with 30 nm spikes (VLPSi-30) and MDA CM-coated VLPSi (CM@VLPSi-30). (D) Relative hydrodynamic diameter (*D*/*D*_0_) of VLPSi and CM-coated VLPSi (CM@VLPSi) in PBS in 1, 2, 4, and 24 h time points. (E) Western blot analysis for HLA-F (MHC-I) and ICAM-1. Ponceau S-stained SDS–PAGE shows the total protein loading and protein bands for CM lysates from (1) MDA and (2) IFN-γ-stimulated MDA (γCM) and VLPSi-30 coated with (3) MDA-CM and (4) γCM. Samples were run at an equal protein concentration (15 μg). (F) Representative confocal image showing the co-localization (white arrows) of VLPSi (green) and CM (red) upon cellular uptake in THP-1 cells. (G and H) Bar graphs showing the biocompatibility (viability) of (G) MDA and (H) MCF-7 cell lines after 24 h of co-culturing with 13, 25, 50, 100, and 200 μg/mL VLPSi-30 with and without CM and γCM coatings. Data are shown as the mean ± SD (*n* = 3). (∗∗∗) *p* < 0.001, (∗∗) *p* < 0.01, and (∗) *p* < 0.05. Scale bars: (C) TEM, 100 nm; (F) confocal images, 10 μm.

To further investigate the distribution within a heterogeneous immune cell population, VLPSi-30 were incubated with PBMC and the impact of CM coatings was assessed using CMs derived from MDA (CM) and IFN-γ-treated MDA cells (γCM). In this context, CD14^+^ monocytes dominated the uptake regardless of the CM source, whereas NK cells consistently showed lower uptake (Figure 1B). This observation goes together with the known phagocytic capacity of monocytes and indicates that VLPSi primarily interact with myeloid cells within PBMCs. Notably, nanoparticle uptake by NK cells was higher in the PBMC context than in expanded NK cells (exNKs), suggesting that NK–nanoparticle interactions could be influenced by the NK cell activation state or cellular microenvironment.

Transmission electron microscopy (TEM) characterization of VLPSi-30 shows their virus-like morphology, featuring surface spikes and an overall diameter of approximately 80 nm (Figure 1A; VLPSi-30). TEM images of CM-coated VLPSi-30 (CM@VLPSi) confirmed the presence of CM on the particles, indicating successful membrane coating (Figure 1C; free CM shown in Figure S2B). CM coating improved colloidal stability in PBS compared to bare VLPSi-30 (Figure 1D). Bare VLPSi-30 aggregated rapidly, whereas the diameter of CM@VLPSi remained stable over 24 h, indicating suitability for biological applications. Preservation of membrane proteins during the coating process was confirmed using SDS–PAGE (Figure 1E and Figure S1D) and western blotting (Figure 1E). IFN-γ-treated MDA was included to the study to modulate MHC-I expression on MDA cells and assess its impact on NK cell activation. Results show that CM and γCM-coated VLPSi-30 preserved their distinct protein bands during the CM-coating process, including IFN-γ-associated upregulation of MHC-I marker (HLA-F) and ICAM-1, seen in free γCM and γCM-coated VLPSi samples (lanes 3 and 5; Figure 1E). The co-localization of VLPSi-30 (green) and CM (red) upon cellular uptake was confirmed using confocal imaging (Figure 1F).

Biocompatibility was assessed using MDA and MCF-7 breast cancer cells. Cells were incubated with bare VLPSi-30 and CM-coated VLPSi-30 (CM@VLPSi-30 and γCM@VLPSi-30) in a concentration-dependent manner for 24 h. Bare VLPSi-30 did not show any detectable impact on cell viability even at the highest concentration of 200 μg/mL (Figure 1G and H), consistent with the known safety profile of mesoporous silica.^20^ In contrast, CM@VLPSi-30 modestly reduced viability in MDA cells (Figure 1F), reflecting the homotypic cell membrane targeting, while γCM@VLPSi-30 caused a 20% decrease in cell viability at the highest concentrations compared to bare VLPSi-30. These results suggest that IFN-γ-induced alterations in CM composition could enhance homotypic interactions without causing off-target toxicity. Together, these structural and biochemical characteristics confirmed the presence of CM on VLPSi, supporting their use in subsequent immunotherapy applications.

NK cell activation requires stimulatory signals, such as interleukin 15 (IL-15), that are often absent in resting PBMCs. To evaluate the ability of VLPSi-30 to modulate NK cell activation within a complex immune population, PBMCs were incubated with VLPSi-30 in the presence or absence of IL-15, a cytokine known to support NK cell proliferation and functional activation. The loading efficiency of IL-15 was determined to be 99.9%, with minimal release over 24 h (<1%), indicating stable association with the particle surface (Figure S1C). NK cell activation was assessed by CD69 and CD25 expression using flow cytometry. In physiological conditions, monocytes prime NK cells through co-stimulatory interactions and cytokine signaling, with IL-2, IL-15, and IL-21 being the key mediators for NK cell activation and proliferation.^21^ However, systemic administration of these cytokines is often limited by their off-target toxicity.^22^ Nanoparticle-based strategies could offer a solution for more localized delivery strategies. Previous studies have demonstrated that IL-15-integrated nanoparticles can enhance IFN-γ production and NK cell infiltration at the tumor site.^23^ This is consistent with our findings. In the absence of IL-15, CM-coated VLPSi-30 (CM@VLPSi-30) induced only a modest increase in the CD69^+^ NK cell population compared to bare VLPSi-30 and medium controls, whereas the CD25^+^ NK cell population remained low (Figure 2A), indicating limited activation without IL-15. When IL-15 (10 ng/mL) was supplemented to the medium (+IL-15) or integrated onto VLPSi-30 (IL15@VLPSi-30), both CD69 and CD25 expressions were upregulated across all groups and further increased with CM coating (Figure 2B). These results demonstrate that IL-15 is essential for robust NK cell activation and that VLPSi can serve as a platform for co-delivery of cytokines and CM ligands.

**Figure 2:**
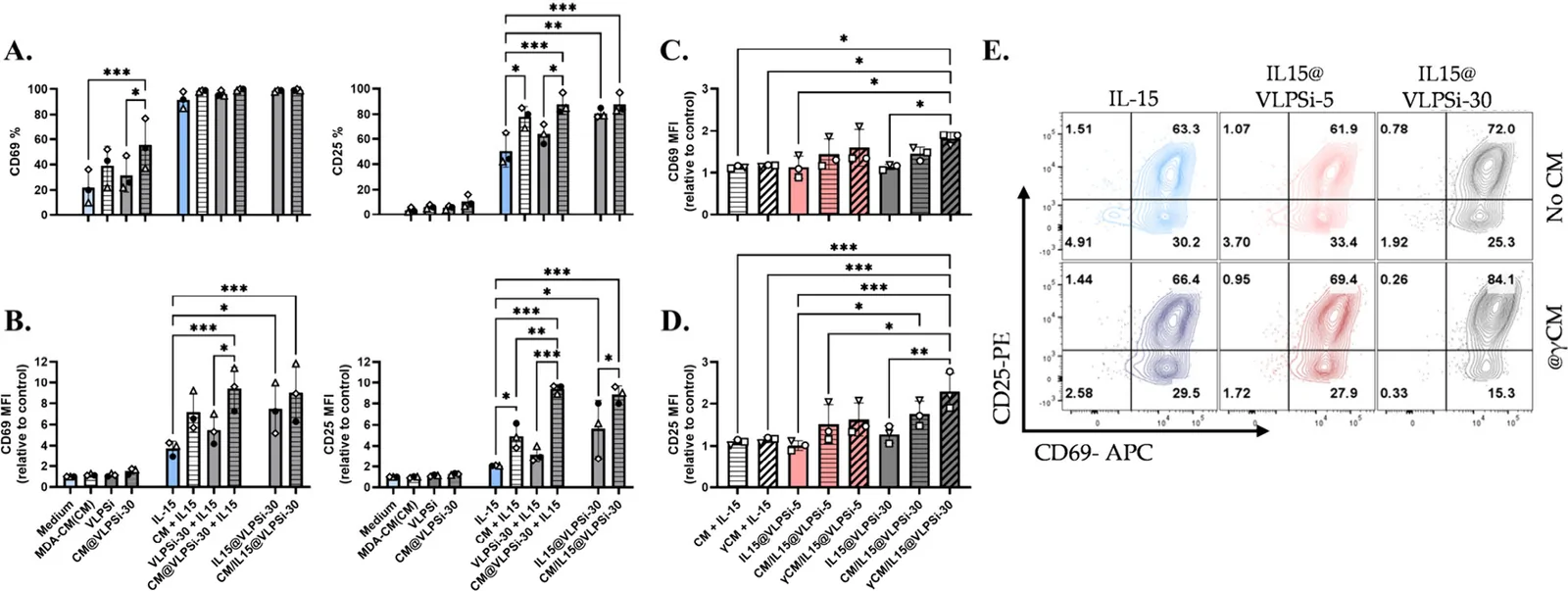
IL-15 and virus-like morphology’s impact on NK cell activation. (A and B) Bar graphs show the (A) percentage of (left) CD69 and (right) CD25 positive NK cells and (B) median fluorescence intensity (MFI) of CD69 and CD25 expression on NK cells normalized to the medium control. Peripheral blood mononuclear cells (PBMCs) were incubated with virus-like mesoporous silica nanoparticles (VLPSi) with 30 nm spikes (VLPSi-30; gray) and MDA-MB-231 CM-coated VLPSi (CM@VLPSi-30; striped) for 24 h with and without 10 ng/mL IL-15 in the media or integrated with VLPSi (IL15@VLPSi). IL-15 (blue) and CM alone (white) were used as controls. (C and D) Bar graphs comparing VLPSi with 5 nm spikes (VLPSi-5; pink) and 30 nm spikes (VLPSi-30; gray) impact to (C) CD69 and (D) CD25 expression with and without CM coating [CM from untreated MDA CM (CM; striped) or IFN-γ-treated MDA CM (γCM; diagonal pattern)] after 24 h of incubation. Free CMs (white) used as controls. (E) Representative contour plot of CD25 and CD69 co-expression on NK cells. Data are shown as the mean ± SD. (∗∗∗) *p* < 0.001, (∗∗) *p* < 0.01, and (∗) *p* < 0.05. Donors are represented by different symbols (*n* = 3).

After the establishment of IL-15 being required for robust NK cell activation, the impacts of MHC-I upregulation and spike length were studied. IL-15-integrated VLPSi with 5 nm (IL15@VLPSi-5) and 30 nm (IL15@VLPSi-30) spikes were coated with CM derived from untreated (CM) or IFN-γ-stimulated MDA (γCM) cells. As previously shown (Figure 1C), γCM has higher MHC-I expressions than standard MDA CM. Across all donors, γCM coating consistently induced strongest NK cell activation. γCM/IL15@VLPSi-30 significantly upregulated CD25 expression compared to bare IL15@VLPSi-5 and IL15@VLPSi-30 and free CM or γCM controls (Figure 2D). CD69 expression followed a similar trend, with γCM/IL15@VLPSi-30 inducing the highest expression (Figure 2C). Co-expression analysis revealed that VLPSi with 30 nm spikes induced the largest population of CD69^+^CD25^+^ NK cells, reaching 84% double-positive cells (Figure 2E). Although higher MHC-I expression is typically associated with NK cell inhibition, γCM coating promoted activation. This suggests that other stimulatory factors present in γCM could override MHC-I-mediated inhibition. IFN-γ treatment has been shown to induce the expression of NK cell activation ligands, such as ICAM-1, on MDA-MB-231 cells,^24^ as was also observed in the WB analysis (Figure 1E), which could outweigh the MHC-I-mediated inhibition.

These results indicate that NK cell activation is influenced by cytokine delivery, CM coating, and spike length, with 30 nm spikes producing the strongest activation. Therefore, VLPSi-30 were selected for all subsequent studies as the most effective platform for the NK cell stimulation.

With the establishment of VLPSi with longer surface spikes (30 nm) enhancing NK cell activation, the influence of surface chemistry was evaluated using baseline MDA CM coating. VLPSi-30 were functionalized with amino (NH_2_), hydroxylamine (NOH), or quaternary amine (^+^N­(CH_3_)_3_, abbreviated as NCH_3_) to investigate how surface chemistry and charge influence NK cell activation. Surface functionalization and membrane coating were assessed by changes in the zeta potential (ζ). Bare VLPSi-30 had a negative charge, which shifted to positive after amino modifications and was restored to a level comparable to free CM after CM coating (Figure 3A), indicating a successful modification and presence of CM on VLPSi-30.

**Figure 3:**
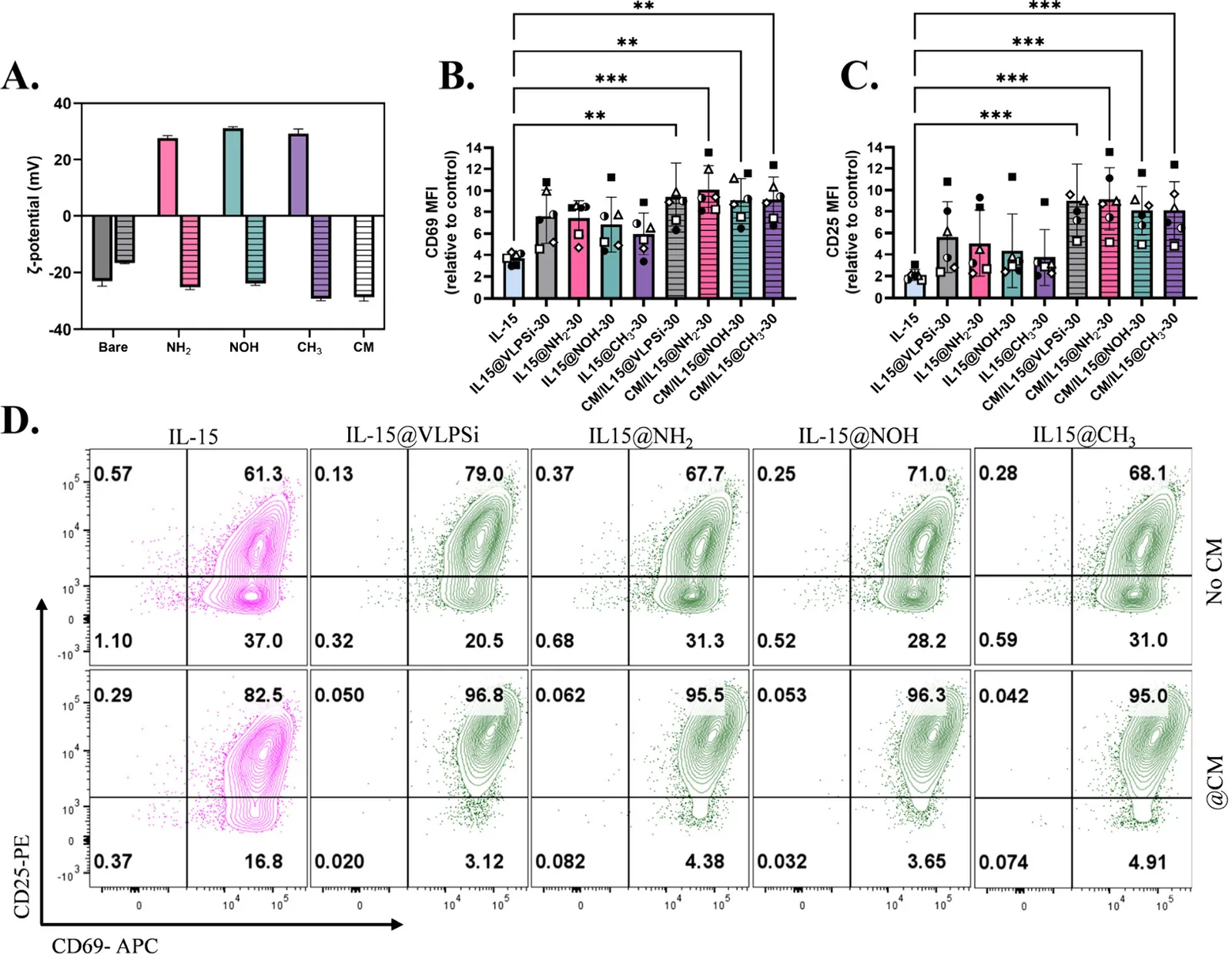
Amino modification impact on NK cell activation. (A) Zeta potential (ζ) of virus-like mesoporous silica nanoparticles (VLPSi) before and after surface modification: unmodified (gray), −NH_2_ (pink), NOH (green), and −CH_3_) (violet) modified, with CM alone and CM-coated VLPSi shown with striped bars. (B and C) Bar graphs show normalized median fluorescence intensity (MFI) of (B) CD69 and (C) CD25 expression on NK cells. Peripheral blood mononuclear cells (PBMCs) were incubated with IL-15-coated virus-like mesoporous silica nanoparticles (IL15@VLPSi-30; gray) or IL15@VLPSi-30 with −NH_2_ (IL15@NH_2_-30; pink), −NOH (IL15@NOH-30; green), and −N­(CH_3_)_3_) (IL15@CH_3_-30; purple) modifications, for 24 h with and without MDA-MB-231 CM coating (striped). IL-15 (blue) and CM alone (white) were used as controls. Donors (*n* = 6) are represented by different symbols. (D) Representative contour plot of CD25 and CD69 co-expression on NK cells, with the first row without CM coating and the second row with CM coating (@CM). Data are shown as the mean ± SD. (∗∗∗) *p* < 0.001, (∗∗) *p* < 0.01, and (∗) *p* < 0.05.

Functionalized VLPSi-30 were evaluated for their ability to activate NK cells within PBMCs from multiple donors. Functionalized VLPSi-30 were subsequently coated with IL-15 (IL15@VLPSi-30) and CM (CM/IL15@VLPSi-30) to assess combinatorial effects of cytokine delivery and CM coating. The NK cell response varied between donors, reflecting the inherent heterogeneity of primary immune cells. Despite this variability, CM coating consistently increased the expression of CD25 (Figure 3C and Figure S4) as well as the CD25^+^CD69^+^ NK cell population compared to controls (IL-15 groups with or without CM; Figure 3D). Among the tested amino modifications, NH_2_-functionalized VLPSi-30 induced the strongest upregulation of both CD25 and CD69, particularly when combined with CM coating (IL15@NH_2_-30; Figure 3B and C). These findings indicate that VLPSi surface chemistry can be fine-tuned to improve NK cell activation, keeping in mind that the strongest effects are achieved through the combination with the CM coating. On the basis of these results, NH_2_-modified VLPSi-30 (NH_2_-30) were selected for the subsequent studies.

Mechanistic studies revealed that NK cell activation was driven primarily by CD14^+^ monocyte–NK cell crosstalk rather than direct VLPSi–NK cell interactions. Within PBMC, monocytes showed high uptake compared to minimal uptake by NK cells (Figure 1B), indicating that monocytes act as the primary sensors of VLPSi. Robust NK cell activation was observed within PBMC, where γCM coating significantly upregulated CD25 and CD69 expression and IL15@NH_2_-30 showed significant upregulation in CD69 expression even without CM coating when compared to the IL-15 control (IL-15) (Figure 4A). Consistently, depletion of CD14^+^ cells (Figure S5) markedly reduced NK cell activation, evidenced by lower CD69 and CD25 expression (Figure 4B).

**Figure 4:**
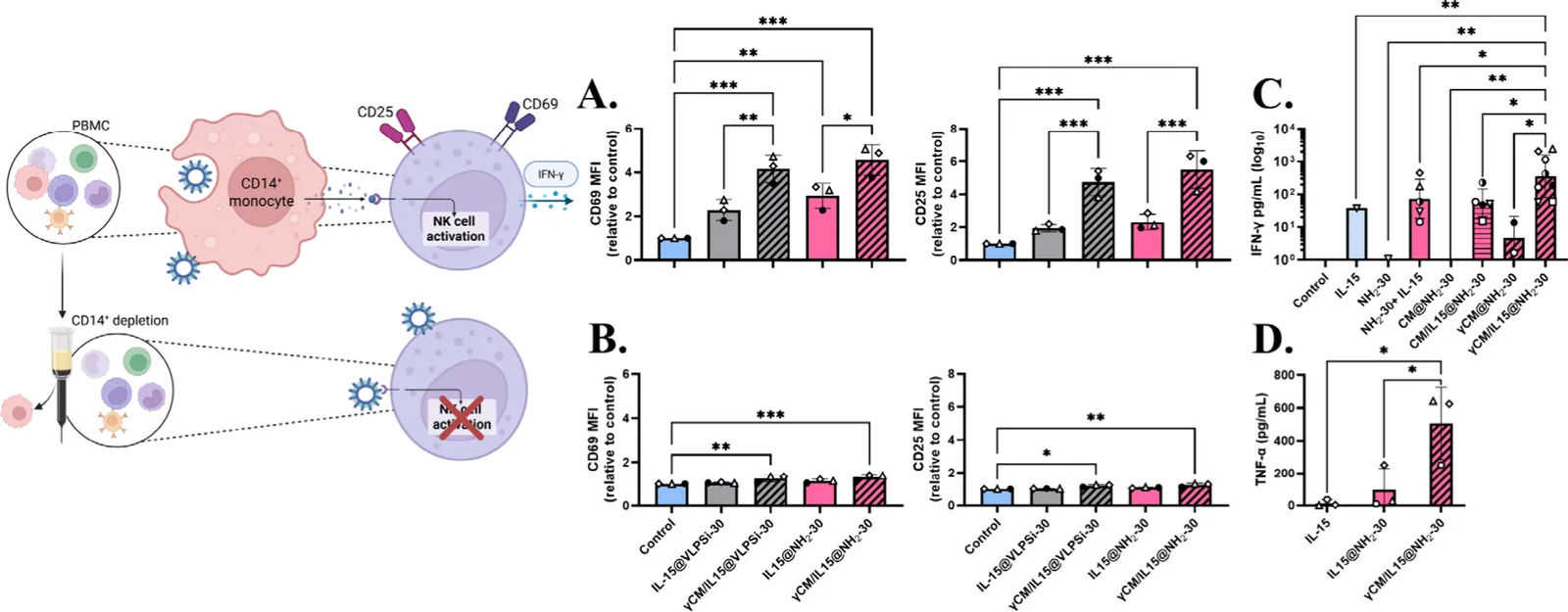
Impact from CD14 depletion. Peripheral blood mononuclear cells (PBMCs) and CD14-depleted PBMCs were incubated with virus-like mesoporous silica nanoparticles with 30 nm spikes (VLPSi-30) incorporated with IL-15 (IL15@VLPSi-30; gray) or −NH_2_-functionalized VLPSi-30 incorporated with IL-15 (IL15@NH_2_-30; pink) for 24 h with and without IFN-γ-treated MDA-MB-231 (MDA) cell membrane (γCM) coating (diagonal pattern). (A and B) CD69 and CD25 expression by normalized median fluorescence intensity (MFI) on NK cells in PBMCs (A) before and (B) after CD14 depletion. *n* = 3. (C) Log_10_-transformed IFN-γ levels (pg/mL) measured from PBMCs incubate with NH_2_-30 with and without IL-15 incorporation and CM or γCM coating. *n* = 8. (D) TNF-α levels (pg/mL) measured from PBMCs incubated with VLPSi-NH_2_-30 with IL-15 incorporation (IL15@NH_2_-30) and γCM coating (γCM/IL15@NH_2_-30). *n* = 3. CMs were derived from MDA (CM; stripes) and γCM (diagonal pattern). Data are shown as the mean ± SD. (∗∗∗) *p* < 0.001, (∗∗) *p* < 0.01, and (∗) *p* < 0.05. Donors are represented by different symbols.

Monocyte-dependent activation was further supported by IFN-γ secretion, which was significantly elevated in PBMC cultures in the presence of IL-15 and below the detection limit after CD14^+^ depletion. The highest IFN-γ levels were observed with IL15@NH_2_-30, particularly when combined with γCM coating (IL15/γCM@NH_2_-30; Figure 4C). To further investigate monocyte-associated cytokine signaling, IL-12p70 (IL-12), IL-18, and TNF-α secretion were measured after incubation with IL-15, IL15@NH_2_-30, or γCM/IL15@NH_2_-30. IL-12 and IL-18 were below the detection limit in all samples. In contrast, TNF-α was detectable in PBMC cultures, with the highest levels observed with γCM/IL15@NH_2_-30, whereas it remained below the detection limit after CD14^+^ depletion (Figure 4D). These results support a monocyte-dependent inflammatory response characterized by TNF-α production and are consistent with the observed IFN-γ secretion and NK cell activation results.

These results indicate that downstream NK cell responses arise indirectly through monocyte-mediated signaling rather than direct contact with VLPSi. This interpretation aligns with a recent proteomics-guided mechanistic study demonstrating spike-length-dependent activation of innate immune signaling pathways and increased inflammatory cytokine expression in murine macrophages, supporting enhanced innate immune sensing of virus-like topologies.^19^ Consistent with our findings, previous studies have shown that depletion of CD14^+^ cells reduces NK cell IFN-γ secretion due to the loss of monocyte-derived cytokine signaling,^25^ supporting our observation that monocytes are critical for NK cell activation. Collectively, these observationsposition monocyte activation as the primary trigger of VLPSi-mediated NK stimulation in PBMCs. These findings are consistent with the known immune cell biology: monocytes function as professional phagocytes and regulators of innate immune communication,^26^ whereas NK cells typically engage their targets through transient immune synapses rather than endocytic pathways.^27^

Engineered K562 cells are widely used in feeder-cell-based NK cell expansion^28^ and have been used to produce nanoscale plasma membrane vesicles, which have been shown to activate NK cells *ex vivo*, and *in vivo*.^29^ These cells present membrane-bound activating ligands capable of directly engaging NK cell receptors. We hypothesized that incorporating CM derived from K562 feeder cells onto VLPSi-30 could enhance NK receptor engagement, potentially strengthening their activation and *ex vivo* expansion even in the absence of accessory monocytes. Purified naïve NK cells were incubated with CM-coated IL15@NH_2_-30. CMs were derived from γCM and genetically engineered K562 feeder cell variants: unmodified K562 cells (K562A), cells engineered to express membrane-bound IL-15 and 4-1BB ligand (K562B), and membrane-bound IL-21, CD48, and 4-1BB ligand (K562C), commonly used in NK cell *ex vivo* expansion.^30^ Naïve NK cell activation remained low under all conditions, except K562C-CM coating, which significantly increased the CD69 expression (Figure 5A) in all donors, while CD25 expression remained low across all conditions (Figure 5B). CD69 is an early activation marker and readily responds to receptor-mediated stimulation, including IL-15 and co-stimulatory ligands, such as 4-1BBL and CD48, present on K562C-derived CM. In contrast, CD25 expression remained low across all conditions within the 24 h assay, indicating that CD25 was not strongly induced under these conditions. This likely reflects the requirement for integrated activation signals for robust CD25 induction, including accessory cell-mediated signaling that is absent in the purified NK cells.

**Figure 5:**
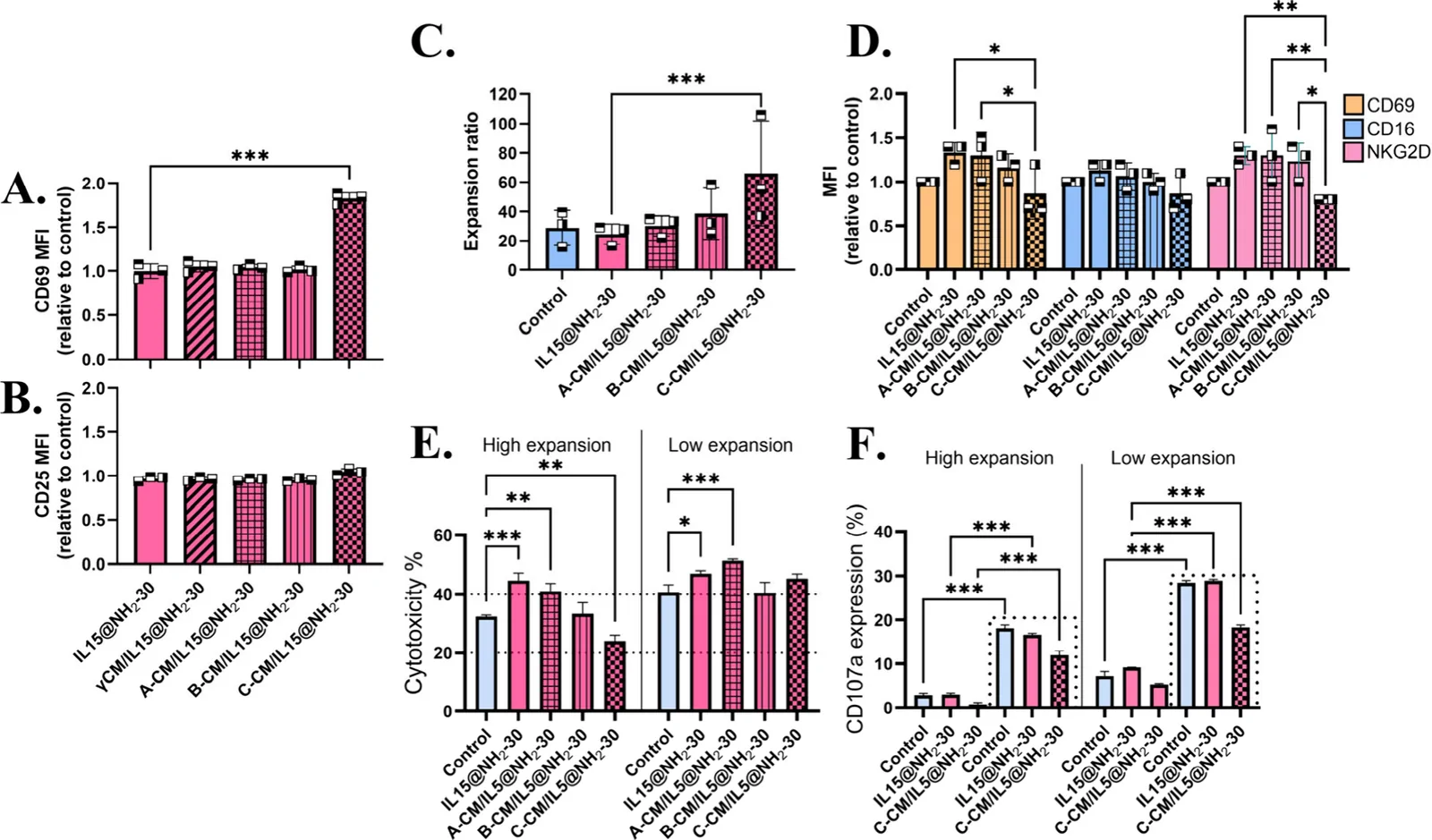
Cell membrane (CM)-coated virus-like mesoporous silica nanoparticles (VLPSi) impact on NK cell expansion and functionality. (A and B) Naïve NK cells were activated with NH_2_-modified VLPSi incorporating IL-15 (IL15@NH_2_-30), with or without CM coating. CMs were derived from unmodified (A; grid) and genetically modified K562 cells expressing membrane-bound IL-15 and 4-1BB ligand (B; stripes) and membrane-bound IL-21, CD48, and 4-1BB ligand (C; checkered). (A) CD69 expression and (B) CD25 expression are shown as normalized median fluorescence intensity (MFI) in naïve purified NK cells after 24 h of incubation. (C) NK cell expansion ratio. Naïve NK cells were expanded for 17 days in media supplemented with IL-2 and IL-15 and activated on days 3 and 10 with IL15@NH_2_-30, A-CM/IL15@NH_2_-30, B-CM/IL15@NH_2_-30, or C-CM/IL15@NH_2_-30. (D) Normalized MFI expression of CD69 (yellow), CD16 (blue), and NKG2D (pink) on NK cells after expansion. (E) Cytotoxicity of the highest and lowest NK cell expansions with K562 CM-coated VLPSi against MDA-MB-231 cells at a 10:1 E/T ratio. (F) CD107a expression in the highest and lowest NK cell expansions with K562C-CM-coated VLPSi, with and without co-culturing with MDA-MB-231 cells (dashed border). E/T = effector to target. Data are shown as mean ± SD. (∗∗∗) *p* < 0.001, (∗∗) *p* < 0.01, and (∗) *p* < 0.05. Donors are represented by different symbols (*n* = 3).

Based on the enhanced CD69 expression observed with K562C-CM coatings, their potential to induce NK cell *ex vivo* expansion was evaluated. CM coating could introduce immune co-stimulatory mimicking ligands, without the need for intact feeder cells, and could provide a safety advantage for NK cell expansion.^31^ Used CMs were derived from K562A (A-CM), K562B (B-CM), and K562C (C-CM) and coated to IL15@NH_2_-30. Naïve NK cells were expanded with the well-established NK cell expansion protocol, in which IL-15 and IL-2 were included during expansion, with feeder cells replaced by CM-coated IL15@NH_2_-30. NK cells were expanded for 17 days, after which the yield, phenotype, and functionality were assessed. The NK cell yield reflected the results of the short-term activation assay (Figure 5A) and K562 CM coating showed promise in NK cell *ex vivo* expansion studies. K562C-CM-coated IL15@NH_2_-30 (C-CM/IL15@NH_2_-30) induced the highest fold expansion (66 ± 36), exceeding expansion rates from the cytokine only control (29 ± 7), bare IL15@NH_2_-30 (25 ± 7; IL15@NH_2_-30), and K562A (30 ± 7; A-CM/IL15@NH_2_-30) or K562B (39 ± 17; B-CM/IL15@NH_2_-30) CM-coated IL15@NH_2_-30 (Figure 5C). These results indicate that membrane composition influenced NK cell proliferation, consistent with the presence of activating ligands (IL-21, CD48, and 4-1BB) in K562C-derived CM. This expansion approach could be further developed by incorporating additional monocyte-derived signals, such as IL-18, onto the VLPSi surface, which have been shown to be advantageous in NK cell expansions.

NK cell activation markers (CD69, CD25, CD16, and NKG2D) were assessed following expansion. CD25 expression was downregulated in all conditions (data not shown), which is a known effect from the use of IL-2 during NK cell expansion.^32^ CD69 and NKG2D expressions were significantly lower in NK cells expanded with K562C-CM-coated IL15@NH_2_-30 (Figure 5D; C-CM/IL15@NH_2_-30), which also induced the highest proliferation rates. This phenotype suggests a shift toward an expansion-dominant state, aligning with previous reports that strong proliferative stimuli can differentially modulate activating receptor expression during *ex vivo* expansion.^32,33^

To determine whether these phenotypic changes were associated with functional alterations, NK cell cytotoxicity was evaluated in donors representing the highest and lowest expansion levels with C-CM/IL15@NH_2_-30 against MDA cells at a 10:1 E/T ratio. NK cells exhibiting the highest expansion showed reduced cytotoxicity, whereas NK cell conditions associated with lower expansion had higher cytotoxicity (Figure 5E). Expansion with uncoated and K562A-CM-coated IL15@NH_2_-30 (A-CM/IL15@NH_2_-30) increased CD69 and NKG2D expression (Figure 5D), and enhanced NK cell cytotoxicity (Figure 5E) compared to the cytokine control. Consistent with the cytotoxicity data, CD107a expression was increased after co-culturing with MDA cells and was highest in NK cells with the lowest expansion, while NK cells with the highest expansion showed lower degranulation activity (Figure 5F). These findings suggest that distinct CM compositions could be used to differentially modulate NK cell activation and function *ex vivo*.

This inverse relationship among expansion, activation marker expression (CD69 and NKG2D), and cytotoxic function is consistent with previously reported trade-offs in NK cell biology, where strong proliferative stimuli can drive cells toward an expansion-dominant but functionally attenuated phenotype. Together, these findings demonstrate that CM-coated IL15@NH_2_-30 enables NK cell expansion and modulates effector function in a CM-dependent manner, providing a strategy to optimize NK cell products for therapeutic applications.

The results established CM-coated VLPSi as a tunable platform in which virus-like topology, surface chemistry, cytokine presentation, and membrane composition together can be used to shape the immune response. CMs derived from different origins could be used as a powerful tool for incorporating specific immunomodulatory signals into the nanomaterial design. This work provides a platform for nanomaterial design that can engage innate immune crosstalk to modulate NK cell activity and holds potential for NK-cell-based cancer immunotherapies.

## Supplementary Material



## Notes

Additional experimental details and materials and methods, including supporting figures (Figures S1–S5): Figure S1, CM-coating optimization results; Figure S2, NK cell activation induced by CM-coated virus-like mesoporous silica nanoparticles (CM@VLPSi-NH_2_); Figure S3, TEM images; Figure S4, representative histogram of CD69 and CD25 expression on NK cells after 24 h of incubation with different amino modifications (VLPSi-30); and Figure S5, representative co-expression plots of CD25 and CD69 expression on NK cells after 24 h of incubation with VLPSi or VLPSi-NH_2_ with and without CM coating (PDF)
